# Machine-Learning-Based Rehabilitation Prognosis Prediction in Patients with Ischemic Stroke Using Brainstem Auditory Evoked Potential

**DOI:** 10.3390/diagnostics11040673

**Published:** 2021-04-08

**Authors:** Jangjay Sohn, Il-Young Jung, Yunseo Ku, Yeongwook Kim

**Affiliations:** 1 Interdisciplinary Program in Bioengineering, Graduate School, Seoul National University, Seoul 03080, Korea; jjaysohn@melab.snu.ac.kr; 2 Department of Rehabilitation Medicine, Chungnam National University College of Medicine, Daejeon 35015, Korea; 102onez@hanmail.net; 3 Department of Biomedical Engineering, Chungnam National University College of Medicine, Daejeon 35015, Korea

**Keywords:** ischemic stroke, brainstem auditory evoked potential, artificial neural network, support vector machine, prognosis

## Abstract

To evaluate the feasibility of brainstem auditory evoked potential (BAEP) for rehabilitation prognosis prediction in patients with ischemic stroke, 181 patients were tested using the Korean version of the modified Barthel index (K-MBI) at admission (basal K-MBI) and discharge (follow-up K-MBI). The BAEP measurements were performed within two weeks of admission on average. The criterion between favorable and unfavorable outcomes was defined as a K-MBI score of 75 at discharge, which was the boundary between moderate and mild dependence in daily living activities. The changes in the K-MBI scores (discharge-admission) were analyzed by nonlinear regression models, including the artificial neural network (ANN) and support vector machine (SVM), with the basal K-MBI score, age, and interpeak latencies (IPLs) of the BAEP (waves I, I–III, and III–V). When including the BAEP features, the correlations of the ANN and SVM regression models increased to 0.70 and 0.64, respectively. In the outcome prediction, the ANN model with the basal K-MBI score, age, and BAEP IPLs exhibited a sensitivity of 92% and specificity of 90%. Our results suggest that the BAEP IPLs used with the basal K-MBI score and age can play an adjunctive role in the prediction of patient rehabilitation prognoses.

## 1. Introduction

In terms of rehabilitation in patients with stroke, the decision making relating to “how to” and “how long” is quite challenging because accurate prognosis of the outcome remains difficult [1]. The clinical need for the prediction of rehabilitation outcomes in patients with stroke is constantly increasing [2]. If it were possible to predict the degree of recovery, a more appropriate treatment strategy and a reasonable rehabilitation goal could be planned according to the patient’s condition [3].

Evoked potentials (EPs) have been widely applied in assessing sensory and motor organs, as well as afferent neural pathways, not only for clinical diagnosis but also for intraoperative neurophysiology monitoring [4]. Moreover, EPs can be used for postoperative assessment to provide prognostic information on clinical outcome and surgical procedure [5,6]. EPs are the electrical signals generated by the nervous system, which respond to external stimuli such as visual, auditory, motor, or somatosensory stimuli. The measurement of EPs does not require large equipment and can be performed minimally or noninvasively. Thus, several studies have investigated the changes in EPs according to the outcome of patients with stroke [7,8,9,10]. Steube and colleagues found that patients with loss of motor evoked potentials (MEPs) from the anterior tibial muscle had lower Motricity Index (MI) scores and reduced rehabilitation effects than those with preserved MEP [10]. Additionally, MEP has also been used as a motor excitation threshold for personalized treatment when applying transcranial magnetic stimulation to improve stroke recovery [11].

However, human studies using the EP for predicting the rehabilitation outcome in patients with stroke are scant. Rollnik evaluated the median somatosensory EP (SEP), brainstem auditory EP (BAEP), and visual EP (VEP) to predict good and poor outcomes, which were divided by a Barthel index (BI) of 50 at discharge [12]. The BI is an ordinal measure for assessing functional dependence in activities of daily living (ADL). The study found substantial differences in the median SEP, latency of wave III in the BAEP, and flash-VEP between two groups in the data recorded at admission. Su et al., focused on the prognosis in patients with severe stroke (Glasgow Coma Scale (GCS) ≤ 12) [3]. The modified Rankin scale, which is another index measuring disability in ADL, was used for favorable (0–4) and unfavorable (5–6) outcomes after six months from the measurements of the GCS, median SEP, and BAEP. The authors found that the GCS score, absence or abnormality of N20 response in the median SEP, and wave V in the BAEP were correlated with the unfavorable outcome. Although BAEP has received relatively little attention in the prognosis of patients with stroke, in their study, the prognostic accuracy using wave V of the BAEP was considerably high (even higher than the median SEP) for the unfavorable outcome (97.5%). It should be noted that 92 out of all 100 patients were considered as having an unfavorable outcome in their study. Consistently, we found a significant delay in the latency of wave V with lower stroke-related clinical assessment scales in our previous study [13]. We hypothesized that the BAEP changes are due to the top-down modulation of brainstem activity by cortical processing [14,15], and thus BAEP could be exploited as a rehabilitation prognostic feature in patients with ischemic stroke even on the infratentorial or supratentorial regions.

Machine learning algorithms have been actively applied to predict the recurrence and survival of various diseases [16,17]. The present study focused on the feasibility of BAEP- and machine-learning-based prognosis in the Korean version of the modified Barthel index (K-MBI) [18,19] of patients with ischemic stroke, which is the most common stroke type (approximately 87%) [20]. First, regression analysis was performed on the change in the K-MBI score (discharge–admission) to investigate the relationships with the basal K-MBI score, age, and interpeak latencies (IPLs) of the BAEP. Age was included because it has been demonstrated that age affects the BAEP latencies [21]. Subsequently, we applied machine-learning-based classification models to evaluate the feasibility in the prediction of favorable or unfavorable outcomes and thereafter observed the changes in the prognostic performance with varying model inputs.

## 2. Materials and Methods

### 2.1. Subjects and Study Protocol

**Figure 1:**
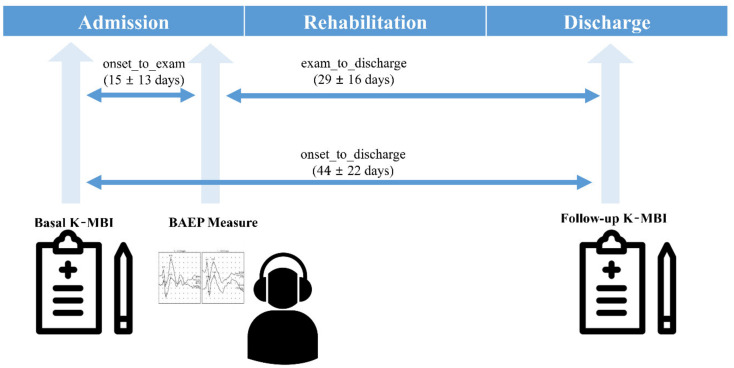
Protocol of the present study.

This study followed the Declaration of Helsinki in medical research and was approved by the Institutional Review Board of Chungnam National University Hospital, Republic of Korea (IRB No. 2019-08-014; 21 August 2019). As this study was a retrospective medical record study, informed consent was waived by the Ethics Committee, which approved the study. A total of 181 subjects with ischemic stroke were screened among patients who were treated in a regional rehabilitation center from May 2018 to April 2019. Included subjects were older than 18 years at the onset of stroke and had suffered an ischemic stroke with corresponding lesions and/or evidence of acute arterial occlusion on MRI. Patients who had a hemorrhagic stroke or other neurological diseases, such as brain tumor, Parkinsonism, or Guillain–Barre syndrome confirmed by MR images and clinical symptoms, were excluded to increase the homogeneity of the target population. Patients with hemorrhagic transformation or recurrence of stroke in admission were excluded, and no other complications that could affect BAEPS were found. Information on number of lesions, extent, and clinical manifestations was not collected. Although the degrees of hearing loss were not confirmed by the pure tone test, patients with severe hearing impairment after stroke were not included in the present study. All subjects were able to communicate with clinicians and clinical pathologists during the rehabilitation process, without auditory assistance. Details of the subject characteristics are presented in Table 1. All subjects received a proper rehabilitation program, such as goal-oriented physical therapy, occupational therapy, and daily activity training of 2 to 3 h per day during their hospital stay. The mean duration of the rehabilitation program was 44 ± 22 days (8 to 114 days) depending on the patient’s condition. They underwent two K-MBI tests, one at admission (basal K-MBI) and another at discharge (follow-up K-MBI). The BAEP measurements were performed 15 ± 13 days (3 to 76 days) following admission (Figure 1).

### 2.2. BAEP Measurement

The BAEP is the electrical potential generated by the auditory nerve and brainstem within the first 10 ms following an acoustic stimulus. A typical BAEP consists of a series of 5 to 7 vertex-positive waves labeled by Roman numerals (waves I–VII). It is considered that waves I through III are produced by the auditory branch of the cranial nerve VIII and lower, whereas waves IV and V originate from the upper brainstem [22]. The BAEP has been applied extensively for monitoring purposes [23,24] or in the prediction of the natural course of diseases [25,26]. In this study, a commercial and validated EP system (Medelec Synergy, VIASYS Healthcare, Surrey, UK) was used for the BAEP acquisition (4 channels: two actives, one reference, and one ground). The BAEPs were recorded in a supine position, with eyes closed. Subjects wore test headphones and were instructed to remain relaxed and quiet during the recording. Scalp needles were placed with Cz as a reference electrode and Fpz as a ground electrode. Following the skin preparation of both mastoids, surface electrodes (A1 and A2) were attached as active electrodes. We recorded the BAEPs from both sides, but only the data from the affected side were analyzed. The impedance of each electrode was maintained at less than 5 KΩ. A total of 1500 rarefaction click sounds, with a length of 100 μs and an intensity of 75 dB normal hearing level (nHL), were used to induce BAEP. It is noteworthy that the stimulus range of 75 dB nHL used in the present study is a relatively high intensity level that produces a low rate of change of approximately 0.1 to 0.2 ms/10 dB in terms of the slope of the latency-intensity function, which could minimize the effect of variation in hearing levels [22]. The interval between click sounds was 100 ms. The final BAEP waveform was calculated by the ensemble averaging of a total of 1500 trials. The acquired signals were filtered with an analog band-pass filter with a cutoff frequency of 3 to 20 kHz, and epochs exceeding ±100 μV were excluded. The BAEP signals with unclear peaks or absence of typical waves (I, III, and V) were excluded from the analysis by eye inspection and automated peak detection of the measurement equipment.

### 2.3. K-MBI

The MBI is used extensively in Europe and Asia for performance measurement in ADL [15]. The MBI is a five-step scoring system that assesses several ADL subtasks, such as personal hygiene, self-bathing, feeding, toilet use, stair climbing, dressing, bowel control, bladder control, ambulation, and chair–bed transfer. The K-MBI has been developed in Korea, and its reliability and validity have been verified [27]. In the K-MBI, a higher score indicates that patients can perform daily life activities with a higher degree of independence. The index includes five categories relating to daily life independence: totally dependent (0–24), severe (25–49), moderate (50–74), mild (75–90), and minimal (91–99). The K-MBI is well suited to regression analysis because its scoring system has 100 levels, whereas there are only seven levels in the modified Rankin scale that was used as an outcome index in another study [3].

### 2.4. Data Analysis

First, to determine the effects of stroke onset (first ever vs. recurrent), lesion location (brainstem vs. other locations), duration from onset to study, and duration from onset to discharge on the BAEP IPLs, we performed partial correlation analysis between each factor and BAEP IPLs while controlling the effect of other factors.

Second, we also performed regression analysis on the change in the K-MBI score (delta K-MBI) using the basal K-MBI score, age, and three IPLs (I, I–III, and III–V) of the BAEP. Two nonlinear regression models using an artificial neural network (ANN) and the support vector machine (SVM) were applied. ANN and SVM regression were employed because these have exhibited high performance without overfitting in many clinical applications [28,29]. In the regression analysis, the radial basis function was used as a kernel function with a regularization parameter (C) of 5 and a gamma parameter of 0.1. As a preprocessing step, Gaussian normalization was applied to the input parameters (basal K-MBI score, age, and IPLs) to estimate the relative feature importance. The correlation coefficients were calculated with *p*-values. Moreover, we assigned a weight to each input parameter and trained the weight vector using a gradient ascent method of the k-nearest neighbors algorithm. The parameter weights were compared to estimate the relative importance in the regression of delta K-MBI. We also performed stepwise regression and observed the change of adjusted *r*-squares to confirm the significance of BAEP IPLs as input variables.

Thereafter, we applied ANN and SVM with the basal K-MBI score, age, and three IPLs to predict the rehabilitation prognosis, which was divided into favorable and unfavorable outcomes. The ANN and SVM have also been widely used for classification tasks [30,31,32]. In this study, K-MBI scores of 0 to 74 (worse than mild dependence) were considered as the unfavorable outcome, whereas K-MBI scores of 75 to 90 (mild and minimal dependencies) were considered as the favorable outcome [33]. The SVM algorithm generates a marginal hyperplane to separate the labeled data in a training session [34]. In the classification, the radial basis function was used as a kernel function with a regularization parameter (C) of 2.76 and a gamma parameter of 0.01. A feed-forward model of the multilayer perceptron neural network classifier was designed for the ANN. This network model included five inputs (K-MBI score, age, and three IPLs) and two outputs (favorable and unfavorable outcomes). In the four-layer structure, the first and second hidden layers included 128 and 64 nodes with the ReLU activation function, respectively. The number of epochs during training was 1000 with a batch size of 50. Approximately 70% of the patients’ data (126 subjects) were used as training data for both models, and five-fold cross-validation was performed. The remaining 30% (55 subjects) were used to test the trained models. The performance in the rehabilitation prognosis prediction was evaluated by the sensitivity, specificity, receiver operating characteristic (ROC) curve, and area under the curve (AUC). The 95% confidence interval was also estimated by the bootstrap method. All statistical analyses and machine learning application processes were performed using MATLAB R2019a (MathWorks, Natick, MA, USA).

## 3. Results

### 3.1. Effects of Confounding Factors on BAEP IPLs

Table 2 presents the results of partial correlation analysis between possible confounding factors and BAEP IPLs. Only a significant but weak correlation was observed between lesion location and IPL of waves III–V (*r* = 0.16, *p* = 0.03). No significant relationship was found between BAEP IPLs and other factors.

### 3.2. Changes in K-MBI Scores

**Figure 2:**
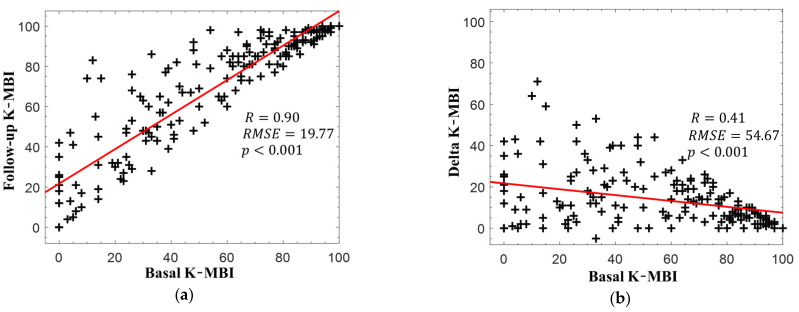
(**a**) Korean version of the modified Barthel index (K-MBI) scores at basal and follow-up examinations and (**b**) changes in K-MBI scores according to initial K-MBI scores. The red lines indicate the linear regression lines.

Figure 2a presents the relationship between the basal and follow-up K-MBI scores. The two scores exhibited a strong correlation (*r* = 0.90, *p* < 0.001), but the relationship between the delta and basal K-MBI scores had a relatively weak correlation (*r* = 0.41, *p* < 0.001), as illustrated in Figure 2b. The scores of most patients increased following rehabilitation, but a higher basal K-MBI score resulted in a lower delta K-MBI score (that is, the ceiling effect). No significant correlations were found between the change in K-MBI score and all intervals (onset to measurement: *r* = 0.09, *p* = 0.19; measurement to discharge: *r* = −0.10, *p* = 0.16; onset to discharge: *r* = 0.21, *p* = 0.07).

**Figure 3:**
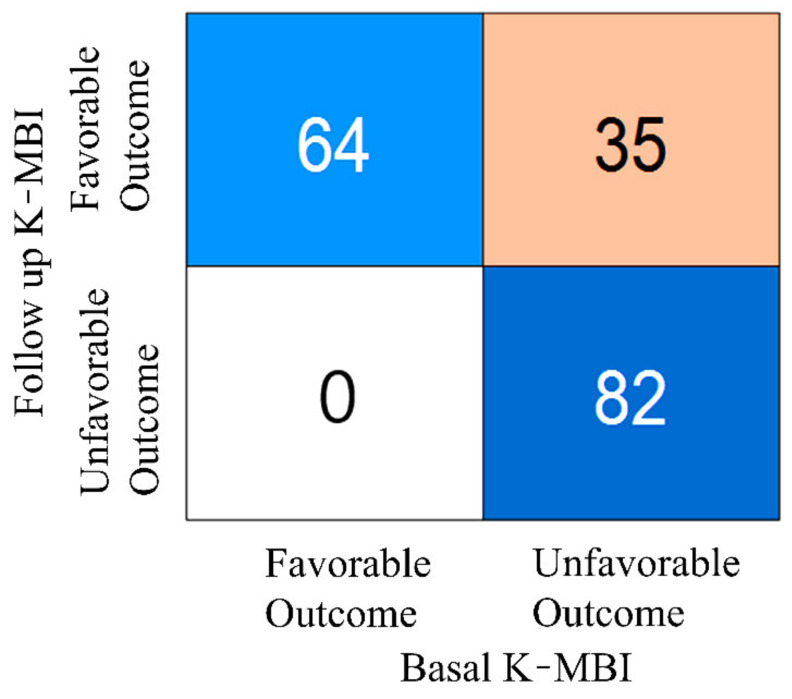
Numbers of patients in favorable and unfavorable groups at basal and follow-up examinations.

The classification result based on the K-MBI score of 74, which was the boundary between mild and moderate dependence, is presented in Figure 3. It is worth noting that the data numbers between the two groups were well balanced. In the training session, the numbers of favorable and unfavorable groups were 69 and 57, respectively. In the test session, the numbers of favorable and unfavorable groups were 30 and 25, respectively. A total of 35 subjects with an unfavorable outcome at the initial examination exhibited a favorable outcome following rehabilitation, indicating that the prediction of the improvement in these patients following rehabilitation was not possible with the basal K-MBI score alone.

### 3.3. Regression and Feature Importance Analysis Subsection

**Figure 4:**
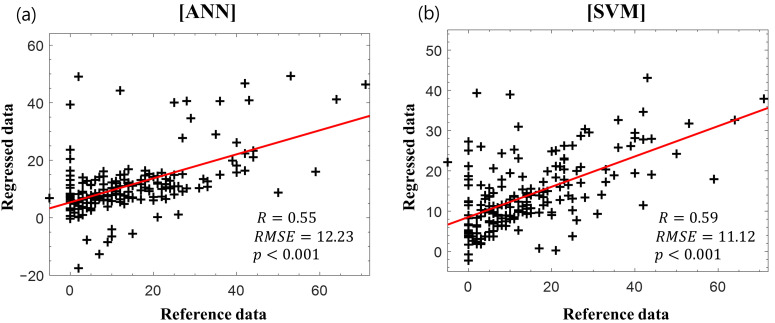
Reference delta K-MBI versus regressed delta K-MBI using (**a**) artificial neural network (ANN) and (**b**) support vector machine (SVM) with the basal K-MBI score and age. The red lines indicate the linear regression lines.

Figure 4 presents the scatter plots of the reference delta K-MBI versus the regressed delta K-MBI when applying (Figure 4a) ANN and (Figure 4b) SVM regressions with the basal K-MBI score and age. Significant correlations were observed in both regression models (ANN: *r* = 0.55, *p* < 0.001; SVM: *r* = 0.59, *p* < 0.001).

**Figure 5:**
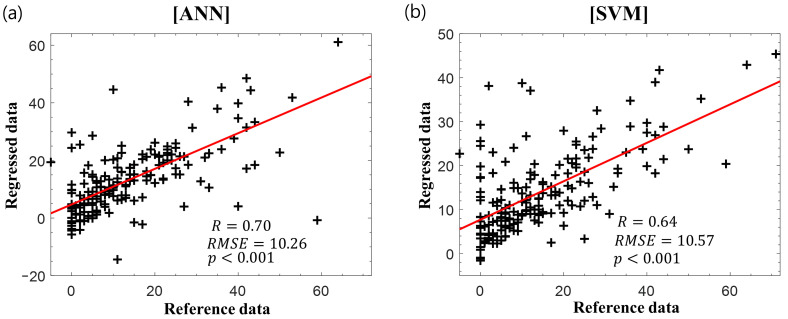
Reference delta K-MBI versus regressed delta K-MBI using (**a**) ANN and (**b**) SVM with the basal K-MBI score, age, and BAEP IPLs. The red lines indicate the linear regression lines.

Figure 5 displays the scatter plots of the reference delta K-MBI versus the regressed delta K-MBI when applying the (Figure 5a) ANN and (Figure 5b) SVM regression with the basal K-MBI score, age, and IPLs of the BAEP. The results of BAEP latencies are listed in Table 2. When incorporating the BAEP IPLs as input variables, the correlations increased in both regression models (ANN: *r* = 0.70, *p* < 0.001; SVM: *r* = 0.64, *p* < 0.001). The weights of all parameters obtained from the k-nearest neighbors algorithm are listed in Table 3. The basal K-MBI score had the greatest importance, whereas the age and latencies of waves I and III–V made considerable contributions to the regression models with similar weight levels. When evaluating the significance of the coefficient of each input variable in the stepwise regression, the basal K-MBI score, age, and latency of waves III–V were significant (*p* < 0.05). The adjusted *r*-squares with different input variables are listed in Table 4. The increase in adjusted *r*-squares was confirmed when incorporating the BAEP IPLs (especially waves III–V) as input variables, which is consistent with the results of ANN and SVM regressions

### 3.4. Prognosis

The test results of the models with the basal K-MBI score and age, as well as the models with the basal K-MBI score, age, and BAEP, are listed in Table 5 and Table 6.

**Figure 6:**
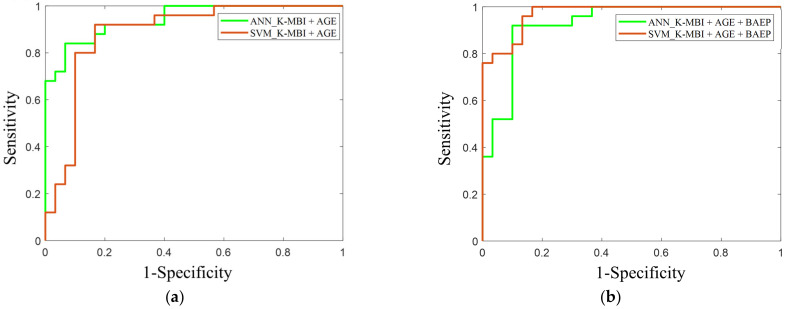
This is a ROC curve in the prediction of favorable and unfavorable outcomes using (**a**) the basal K-MBI score and age, and (**b**) the basal K-MBI score, age, and BAEP IPLs (I, I–III, and III–V). The green lines indicate the ROC curves of the ANN models, and the red lines indicate the ROC curves of the SVM models.

In the ANN model with two input features, the sensitivity and specificity were 84% and 86%, respectively. In the SVM model with the same two inputs, the sensitivity and specificity were 84% and 90%, respectively. When adding the three BAEP IPLs as input features, the sensitivity and specificity in the ANN model increased to 92% and 90%, respectively, whereas those in the SVM model were 88% and 86%, respectively. The AUC values of the ANN and SVM models when using the basal K-MBI score and age were 0.90 and 0.87, respectively. When adding the three BAEP IPLs, the AUC values of the models increased to 0.93 and 0.90, respectively (Figure 6).

## 4. Discussion

The main findings of this study are twofold. First, the BAEP IPLs with a basal K-MBI score and age significantly improved the regression predictive ability on the changes in the K-MBI scores between admission and discharge. Second, the BAEP IPLs also contributed to the prognostic performance of nonlinear machine learning models when predicting the favorable and unfavorable groups with the K-MBI score at discharge.

In the regression analysis of the change in the K-MBI score between admission and discharge, we found that the BAEP IPLs could contribute considerably to the outcome prediction. It has been controversial, but some studies reported that BAEP could be a predictor of the prognosis of patients with supratentorial or infratentorial stroke [35]. A previous study reported a substantially longer latency of wave III in the poor outcome group (BI < 50) [12]. In another study, the poor differentiation or the absence of wave V was correlated with the unfavorable outcome (modified Rankin scale: five or six) [3]. Brainstem lesions are associated with poor neurological outcomes, [36] but the present study included patients with ischemic stroke only on the supratentorial or infratentorial regions, or both. Su and colleagues suggested that BAEP may reflect abnormality regarding the extent of supratentorial brain injury and evolving brainstem compression. Auditory brainstem regions could presumably be sensitive to top-down processes, and thus, lesions on the supratentorial and infratentorial regions could affect the brainstem responses [37,38].

A strong association was observed between the basal and follow-up K-MBI scores, whereas the change in the K-MBI score exhibited a lower dependence on the initial status. Moreover, when analyzing the favorable and unfavorable groups, 30% of patients (35 out of 117) with an unfavorable outcome at admission improved to the favorable group following the rehabilitation process. The ANN model exploiting the BAEP IPLs together with the basal K-MBI score and age could increase the sensitivity and specificity of predicting the unfavorable outcome up to 92% and 90%, respectively. This improvement in the prediction was also clearly confirmed by the ROC curves. It is likely that the neural network model is well suited to estimate the nonlinear relationship of rehabilitation outcomes. This is in line with a previous study, which found that the optimal outcome prediction performance for acute stroke was achieved with the deep neural network. Although different input features such as clinical variables or medication history (a total of 38 features) were applied in the previous study, the deep neural network outperformed other machine learning models such as random forest or logistics regression [39].

The present study exhibits several limitations. The size of the test dataset was relatively small, and the test was performed only in a retrospective nature. The degrees of hearing loss were not confirmed by the pure tone test. The confirmation of the hearing ability can provide more accurate information to differentiate the effect of hearing loss vs. stroke and then to evidence the contribution of BAEP in the prognosis prediction of patients with stroke. Subjects were also limited to patients with ischemic stroke on the supratentorial or infratentorial regions, or both. There was a lack of data on the pathogenesis of ischemic stroke and lesion size. Furthermore, there was a lack of data with large delta K-MBI scores over 50. Additional features from auditory middle-latency and late responses that measure cortical-subcortical functions and multiple measurements could provide more information for the prognosis of patients with stroke. Several studies reported an association between the functional prognosis or poor outcome of stroke patients and multimodal evoked potentials (MEP and SEP), and greater predictability was found when combined as multimodal evoked potentials [40,41]. Similar results were found not only in stroke patients but also in patients with traumatic brain injury or hypoxic brain damage [1,42]. Therefore, the incorporation of additional physiological information, such as SEP or VEP, into the inputs of the machine learning models could improve the rehabilitation prognosis predictability. Further prospective studies with large and heterogeneous patient populations are warranted to develop a prognostic method.

## Figures and Tables

**Table 1:** Characteristics of stroke patients.

Characteristics	Total	
Participants, *n*	181	
Demographics		
Sex, % males	56.35	
Age, years, mean ± SD (min/max)	68.15 ± 11.68 (36/89)	
Stroke onset, *n*		
First ever	142	
Recurrent	39	
Lesion side of cerebral infarction, *n*		
Right	83	
Left	98	
Lesion location, *n*		
Supratentorial		
Cortex	75	
BG/IC	41	
Thalamus	4	
Corona radiata	23	
Infratentorial		
Brainstem	33	
Cerebellum	11	
Both	2	
Duration, days		
From onset to study, mean ± SD	15.82 ± 13.16	
From onset to discharge, mean ± SD	44.04 ± 22.24	
Clinical evaluation level, mean ± SD	At admission	At discharge
K-MBI	53.08 ± 31.71	67.24 ± 30.19
NIHSS	5± 4.69	3.87± 4.28

**Table 2:** Partial correlations between brainstem auditory evoked potential (BAEP) interpeak latencies (IPLs) and possible confounding factors.

Partial Correlations	Stroke Onset	Lesion Location	Onset to Study	Onset to Discharge
**Wave I**				
*r*	−0.02	0.04	0.09	0.07
*p*	0.82	0.61	0.22	0.36
**Waves I–III**				
*r*	−0.13	0.06	−0.07	-0.03
*p*	0.08	0.39	0.38	0.66
**Waves III–V**				
*r*	0.02	0.16	0.05	0.01
*p*	0.82	0.03	0.51	0.97

**Table 3:** Comparisons between lesion and nonlesion sides (mean ± SD).

Input Features		Wave I			Waves I–III			Waves III–V		
BAEP Latency (msec)		L.S (mean ± SD)	N.S (mean ± SD)	*p*-Value	L.S (mean ± SD)	N.S (mean ± SD)	*p*-Value	L.S (mean ± SD)	N.S (mean ± SD)	*p*-Value
Total subjects		1.83 ± 0.21	1.85 ± 0.22	0.37	2.24 ± 0.22	2.25 ± 0.25	0.73	3.83 ± 0.32	3.82 ± 0.29	0.47
Lesion location	Supratentorial	1.83 ± 0.20	1.86 ± 0.23	0.25	2.24 ± 0.24	2.26 ± 0.26	0.25	3.81 ± 0.30	3.82 ± 0.30	0.83
	Infratentorial	1.84 ± 0.23	1.83 ± 0.18	0.85	2.21 ± 0.16	2.19 ± 0.20	0.33	3.89 ± 0.39	3.83 ± 0.26	0.41

Abbreviations: BAEP, brainstem auditory evoked potential; SD, standard deviation, L.S.; lesion side, N.S; nonlesion side.

**Table 4:** Feature importance (KNN and stepwise regression).

Feature Importance	KNN	Stepwise Regression		
	Feature Weight (Arbitrary Value)	Coefficients	*t*-Value	Sig.
K-MBI (basal)	6.48	−6.45	−6.32	2.16 × 10 ^−9^ **
Age	2.28	−4.17	−4.06	7.35 × 10 ^−5^ **
Wave I	2.54	0.21	0.18	0.86
Waves I–III	0.01	0.78	0.55	0.58
Waves III–V	2.64	−2.96	−2.27	0.02 *

(Sig.: significance level, * *p* < 0.05, ** *p* < 0.005).

**Table 5:** Adjusted *r*-squares in stepwise regression.

Input Variables	Adjusted *r*-Square
K-MBI (basal)	0.20
K-MBI (basal) + Age	0.32
K-MBI (basal) + Age + Waves III–V	0.48
K-MBI (basal) + Age + Waves I–III + Waves III–V	0.44
K-MBI (basal) + Age + Wave I + Waves I–III + Waves III–V	0.40

**Table 6:** Test results in prediction of favorable and unfavorable outcomes.

Input Features	Model	Accuracy	Sensitivity	Specificity	AUC
K-MBI (basal) + Age	ANN	85% (0.76 to 0.94)	84% (0.74 to 0.94)	86% (0.77 to 0.95)	0.90 (0.82 to 0.97)
	SVM	87% (0.78 to 0.96)	84% (0.74 to 0.94)	90% (0.82 to 0.97)	0.87 (0.78 to 0.96)
K-MBI (basal) + Age + IPLs (I, I–III, and III–V)	ANN	91% (0.83 to 0.99)	92% (0.84 to 0.99)	90% (0.82 to 0.97)	0.90 (0.82 to 0.97)
	SVM	84% (0.74 to 0.94)	88% (0.79 to 0.96)	86% (0.77 to 0.95)	0.93 (0.86 to 0.99)

The numbers in parentheses indicate 95% confidence intervals.

## Data Availability

Data cannot be shared publicly because of privacy concerns. Data will be made available upon request from the Chungnam National University Hospital Institutional Review Board (+82-42-280-6715) for researchers who meet the criteria for access to confidential data.
